# Balancing Urgency and Strategy in the Surgical Management of a Complex Case of Gallstone Ileus: A Surgical-Video-Based Case Report of a 60-Year-Old Female

**DOI:** 10.7759/cureus.67304

**Published:** 2024-08-20

**Authors:** Hisham Alabdullah, Fayez G Aldarsouni, Hatoon Dagestani, Hassan Mashbari

**Affiliations:** 1 Department of Surgery, King Saud University Medical City, Riyadh, SAU; 2 Department of Trauma Surgery, King Saud Medical City, Riyadh, SAU; 3 Department of General Surgery, Prince Sultan Military Medical City, Riyadh, SAU; 4 Department of Surgery, Al-Iman General Hospital, Riyadh, SAU; 5 Department of Surgery, Jazan University, Jazan, SAU

**Keywords:** surgical videos, case report, general surgery, bouveret's syndrome, mirizzi syndrome, cholecystectomy, emergency surgery, acute care surgery and trauma, gallstone ileus, small bowel enterotomy

## Abstract

Gallstone ileus is an uncommon but potentially life-threatening complication of gallstone disease, characterized by the obstruction of the gastrointestinal tract by a gallstone, typically at the ileocecal valve. This condition predominantly affects elderly patients and carries a high risk of morbidity and mortality due to delayed diagnosis and the complexity of associated comorbidities.

We report the case of a 60-year-old woman with a history of hypertension and cholelithiasis who presented with a four-day history of intermittent epigastric pain, nausea, vomiting, and an inability to pass stool or flatus. Initial imaging studies, including ultrasonography and computed tomography, revealed a biliary-enteric fistula with a large obstructing gallstone at the ileocecal valve. Despite conservative management with intravenous fluids, nasogastric tube suction, and antibiotics, the patient’s symptoms persisted, necessitating surgical intervention. A midline laparotomy was performed, during which the gallstone was successfully removed via enterotomy. The patient recovered without complications and was discharged in stable condition.

The complexity of management, particularly in elderly patients with multiple comorbidities, necessitates careful consideration between the one-stage and two-stage surgical approaches. In this case, the decision to perform an enterotomy without immediate cholecystectomy reflects a two-stage strategy, aimed at minimizing operative risk while addressing the immediate obstruction. This approach underscores the need for individualized management plans, where the choice between one-stage and two-stage surgery is guided by the patient's overall clinical status.

## Introduction

Gallstone ileus, a mechanical impaction of single or multiple stones in the ileocecal valve, is one of the least common complications of cholelithiasis, with an incidence of 0.15%-1.5% [1,2]. Bartholin first described it in the mid-17th century in a necropsy study [3]. In the 1890s, Bouveret reported the first case series of 131 cases of gallstone ileus, with a mortality rate of 44%, highlighting its fatality [4,5]. Mortality rates have reached as high as 60% in old reports [5]. However, these trends have improved over the years due to advancements in diagnostic and management techniques [5-7]. The pathophysiology of gallstone ileus involves adhesions resulting from chronic gallbladder inflammation [2,3]. This inflammatory and adhesive process eventually leads to a biliary-enteric fistula through necrosis and erosion [3,5,7,8]. The surge in gallstone ileus cases could be attributed to the overall increase in the incidence of biliary cholic, which is consequently related to lifestyle changes and the aging population in recent centuries [5,8]. Gallstone ileus predominantly affects females and older adults [9]. Surgical intervention for this disease carries higher risks in the elderly due to higher comorbidity rates contributing to a complex presentation [5,8]. Anticipating postoperative complications becomes a challenge in these patients, as late surgical management is often unanticipated due to the waxing and waning nature of symptoms at presentation, consequencing eventually to bowel necrosis, shock, and peritonitis concurrent with severe dehydration if not managed [2,5,9]. We present a video case report of a 60-year-old female who presented with waxing and waning epigastric pain, discussing the diagnosis and management of the disease.

## Case presentation

A 60-year-old woman with a background of hypertension and cholelithiasis; otherwise, medically and surgically free, presented to the emergency department (ED) complaining of epigastric abdominal pain for four days. In addition to pain, an association with multiple episodes of vomiting was observed, which became brownish and foul-smelling within the last day. She could not tolerate any oral intake, including fluids, over the previous three days but began tolerating small amounts of liquids on the day of the presentation. She also reported not passing stool or flatus for three days. There was no history of fever, diaphoresis, changes in urinary symptoms, loss of consciousness, cough, or shortness of breath. The patient mentioned visiting an outside hospital where she received antiemetics but experienced no improvement in her symptoms despite it.

On examination, she was afebrile and vitally stable, with a heart rate of 108-114 bpm and a blood pressure of 112/88 mmHg. Her abdomen was distended with epigastric tenderness, but there was no rebound tenderness, and Murphy's sign was negative. Digital rectal examination (DRE) revealed an empty rectum.

Laboratory tests were remarkable: white blood cell count of 18,000/mm³, hemoglobin of 16 g/dL, platelets of 391,000/mm³, and creatinine of 255 µmol/L. Liver function tests, electrolytes, and coagulation profiles were within normal limits. See Table 1.

**Table 1:** Lab results taken at the time of presentation in the emergency room WBC: White Blood Cell Count (10^3/uL), Hgb: Hemoglobin (g/dL), Plt: Platelets (10^3/uL), Cr: Creatinine (µmol/L), AST: Aspartate Aminotransferase (U/L), ALT: Alanine Aminotransferase (U/L), BUN: Blood Urea Nitrogen (mg/dL), Bilirubin: Total Bilirubin (mg/dL), PT: Prothrombin Time (sec), aPTT: Activated Partial Thromboplastin Time (sec), INR: International Normalized Ratio

Lab Parameter	Result	Reference Range
White Blood Cell Count	18,000/mm³	4,000-11,000/mm³
Hemoglobin	16 g/dL	(female): 12.0-15.5 g/dL
Platelets	391,000/mm³	150,000 - 450,000 /mm³
Creatinine	255 µmol/L	53-106 µmol/L
BUN	23.90 mg/dL	7 - 20 mg/dL
Bilirubin	0.8 mg/dL	0.1 - 1.2 mg/dL
AST	36.10 U/L	0 - 40 U/L
ALT	40.00 U/L	0 - 40 U/L
Alkaline Phosphatase	70 IU/L	44 - 147 IU/L
PT	17.47 second	11 - 14.5 seconds
aPTT	45.00 second	25 - 35 seconds
INR	1.27	0.8 - 1.2

**Figure 1:**
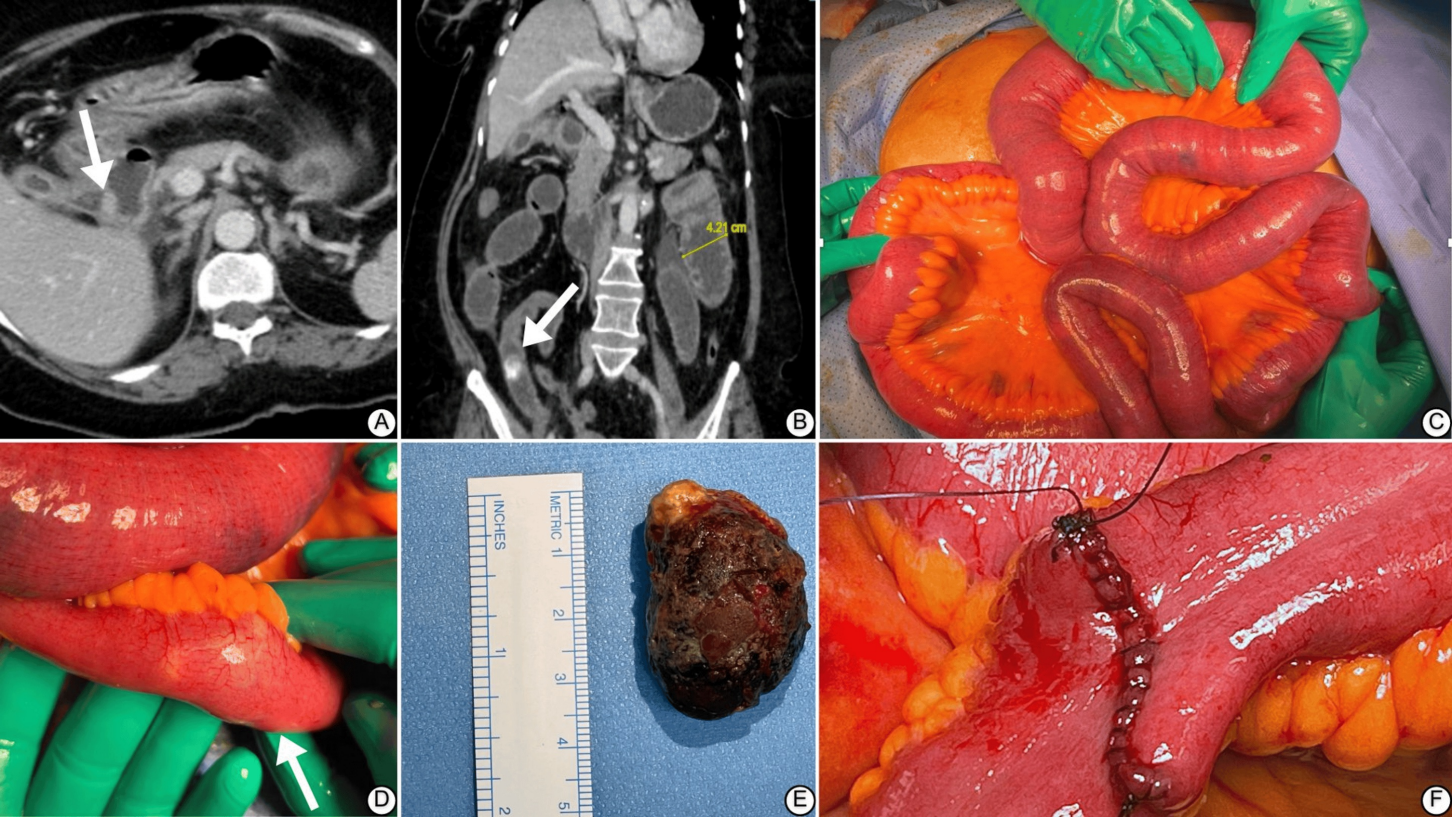
Gallstone ileus – CT images and intraoperative findings (A) The axial CT image shows a dilated gallbladder with an arrow pointing to the site where the gallbladder lumen communicates with the first part of the duodenum. The thickened walls of both the gallbladder and duodenum, along with surrounding fat stranding and free air, are indicative of chronic inflammation and the formation of a biliary-enteric fistula. (B) The coronal CT image highlights a 4.21 cm dilation (yellow line) and the stone (marked by an arrow). The distended loops of the small intestine proximal to the obstruction are visible, along with reactive bowel wall thickening. (C) The surgical field is exposed, showing dilated loops of the small intestine. the small bowel is significantly distended as a result of obstruction. (D) A closer view of the obstructed segment of the small intestine shows a bulge (arrow) where gallstone is identified. The impacted gallstone caused a complete blockage of the bowel. The bowel wall appears thickened and inflamed around the site of the obstruction. (E) The removed gallstone measures approximately 3.5 cm in size, shown next to a ruler for scale, with an irregular, rough surface. (F) The final step of the surgery shows the enterotomy site being closed transversely with continuous 3-0 Vicryl sutures. The closure is reinforced with Lembert continuous sutures using the same material, ensuring a secure and leak-proof seal.

Ultrasonography revealed an inflamed gallbladder. A computed tomography (CT) scan conducted in the ED settings showed that the gallbladder lumen communicated with the first part of the duodenum (Figure 1A), with reactive thickening of the duodenal and gallbladder walls, surrounding fat stranding, and free fluid. The gallbladder and biliary system showed multiple air fluid levels. Abdominopelvic-free fluid was noted, and minor bowel wall enhancement was preserved without evidence of perforation or bowel ischemia. The impression was consistent with gallstone ileus as a stone was observed in the CT (Figure 1B).

The patient was admitted for observation and conservative management, kept on nothing by mouth (NPO), and given intravenous fluids (IVF) with nephrology involvement, attributing the high creatinine level to pre-renal azotemia secondary to the multiple episodes of vomiting. An NG tube was inserted and kept on intermittent suction, and she was administered antibiotics and proton pump inhibitors in preparation for surgical intervention.

The patient was taken to the operating room (OR) as a Category 1 emergency in stable condition. After general anesthesia induction and endotracheal tube insertion, she received 2 grams of cefazolin and 5000 units of subcutaneous heparin. A midline 15 cm incision was created, and the fascia was identified and opened safely. Upon entry, turbid fluid was encountered, cultured, and suctioned. A thorough exploration was done, with the small bowel run from the duodenojejunal junction to the terminal ileum (Figure 1C), where an obstruction was palpated externally due to a stone without any visible external changes (Figure 1D). No additional stones were identified, and the gallbladder and fistula site were not visualized due to edema. The obstructing stone was milked 40 cm above the site of obstruction. A 3 cm longitudinal enterotomy was created along the axis of the bowel, releasing fluid, loose stool, and food content, which were controlled and suctioned outside the laparotomy incision (Video 1). A 3.5 cm stone was retrieved (Figure 1E). The enterotomy was closed transversely with continuous 3.0 Vicryl sutures (Ethicon, Inc., Johnson & Johnson, NJ, USA), reinforced with Lembert continuous sutures using the same material (Figure 1F). Multiple irrigations and suction ensured thorough cleaning, and hemostasis was secured. Local analgesia was administered, the fascia was closed with loop PDS 1-0, and the skin was closed with clips. Dressing was applied, and she was shifted to the post-anesthesia care unit in stable condition after being extubated. She stayed for observation before being discharged home in her usual health. She was followed for 12 months postoperatively without complications. The patient favored not to undergo cholecystectomy, as her symptoms ultimately improved.

**Video 1:** Real-time CT imaging and enterotomy for gallstone ileus: surgical procedure This video documents the surgical procedure performed on a 60-year-old female patient diagnosed with gallstone ileus, as detailed in the accompanying case report. The footage provides a real-time view of the CT scans and the surgical steps.

## Discussion

**Figure 2:**
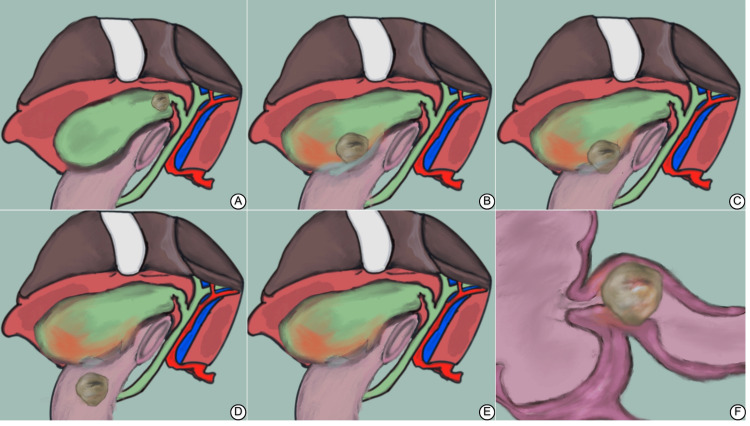
Pathophysiology of gallstone ileus (A) Initially, gallstones form within the gallbladder. The gallbladder appears inflamed, indicating chronic cholecystitis, which is a predisposing factor for the development of gallstones. (B) Due to chronic inflammation, the gallbladder wall thickens, and a fistula begins to form between the gallbladder and the adjacent duodenum. This inflammation is a result of the continued irritation from the gallstones. (C) Over time, a gallstone migrates through the fistula into the duodenum. The figure shows the movement of the gallstone from the gallbladder into the intestinal lumen. (D) The gallstone travels through the intestines and becomes lodged in the ileocecal valve, leading to mechanical obstruction. This is a critical stage where the patient may present with symptoms of bowel obstruction. (E) The impacted gallstone causes a complete blockage of the intestine. The figure highlights the location of the obstruction and the resultant dilatation of the proximal bowel segments. (F) A detailed view of the gallstone lodged at the ileocecal valve, causing an obstruction. Figure credit: Fayez Aldarsouni

Gallstone ileus is a silent killer, striking unexpectedly and demanding immediate surgical action to save lives. The silence of symptoms usually develops into a waxing and waning nature, contributing to the delayed presentation often encountered with this disease [2,5]. The pathophysiology of this condition was a secret until Thomas Bartholin described it in 1654, examining cadavers in an era where mortality from this disease was a striking mystery [3,4]. As highlighted in Figure 2, the hallmark of this disease is the chronic inflammation from an inflamed non-cholecystomized gallbladder [2]. A stone wanders (Figure 1A) between the cystic duct until it settles at one end of the gallbladder. Swelling and inflammation limit its movement, yet the inflammation causes the gallbladder to adhere to the duodenum due to proximity (Figure 2B) [3]. However, there were reports where such a fistula involved the stomach, jejunum, and colon [2]. This inflammation along with ischemia and erosion caused by stone contributes to fistulation (Figure 2C) [7]. The fistula enlarges to accommodate the gallstone into the duodenum (Figure 2D), and the gallstone travels through the duodenum (Figure 2E) until it lodges in the gastric outlet or the proximal duodenum (Bouveret syndrome) or at the narrow ileocecal valve (gallstone ileus) (Figure 2F) [10].

This pathophysiology explains the waxing and waning of symptoms. Cooperman et al., in a review of 15 cases, found an average time from symptoms to presentation of 7 days [6]. In our case, the patient started experiencing symptoms four days before the presentation, making it relatively early compared to the previous report. We attribute this early presentation to the rapid progression of symptoms imposed by a significant 3.5 cm gallstone (Figure 1E). Both the location and size of the stone can be associated with gallstone obstructive diseases [5]. Common symptoms include abdominal pain, distention, opacification, constipation, nausea, and vomiting, but the absence of symptoms doesn't exclude the disease; as many as 30% of patients do not present with biliary symptoms. The character of vomiting can hint at the stone's location [5,9]. When the gallstone is in the stomach or upper small intestine (Bouveret syndrome), the vomitus is mainly gastric content and becomes feculent when the ileum is obstructed (gallstone ileus) [9]. Our patient presented with typical symptoms involving abdominal pain, and the vomiting was characteristic of distal obstruction, "brown in color."

Discussing gallstone ileus raises the thought of other complications of gallstone diseases as differential diagnoses (Table 2). Mirizzi syndrome, hallmarked by a chronic inflammatory process, can progress to a cholecystocholedochal fistula with a pathophysiology similar to what we highlighted in Figure 2. However, this fistula results from a chronic impacted stone in the cystic duct, which, due to proximity to the common hepatic duct, can cause a different presentation [11]. In Mirizzi, symptoms of jaundice appear early, unlike gallstone ileus, a silent disease. Mirizzi often presents with symptoms of obstructive jaundice secondary to compression of the common hepatic duct, causing symptoms far before fistulization occurs. However, this doesn't exclude gallstone ileus or Bouveret syndrome in jaundiced patients, as it may present in 15% of patients with other gallstone obstructive diseases [5].

**Table 2:** Comparative overview of Bouveret syndrome, gallstone ileus, and Mirizzi syndrome

Aspect	Bouveret Syndrome	Gallstone Ileus	Mirizzi Syndrome
Cause	Obstructing gallstone in the gastric outlet/duodenum	Gallstone lodged in the intestine via a fistula	Impacted gallstone in the cystic duct/gallbladder neck
Pathophysiology	Cholecystoduodenal fistula leading to proximal obstruction	Cholecystoduodenal fistula leading to distal obstruction	Compression of the bile duct by the impacted gallstone; Type V involves fistulization and gallstone passage
Symptoms	Gastric outlet obstruction, nausea, vomiting (green)	Bowel obstruction, crampy abdominal pain, vomiting (brown)	Obstructive jaundice, right upper quadrant pain

Moreover, there is a reported case where gallstone ileus and Mirizzi co-occur, as both differentials share common risk factors [7]. Bouveret syndrome is the rarest form of gallstone obstructive disease [12] with a pathophysiology similar to gallstone ileus but differing in the location of the obstruction. Bouveret syndrome stones obstruct the gastric outlet or proximal duodenum, causing symptoms similar to gastric outlet obstruction. It more commonly presents with epigastric pain and early satiety [5]. Bouveret syndrome can progress into a classical gallstone ileus if the stone migrates distally leading to a shift in symptoms [12].

Rigler's triad - a tried that is highly suggestive of gallstone ileus - was observed in our patient on imaging, showing pneumobilia, small bowel obstruction (dilated bowel loop), and ectopic gallstone. The presence of two signs is considered pathognomonic for the disease [5,9]. A CT scan is superior to an abdominal X-ray or ultrasound, with a sensitivity of up to 93%. Although Rigler's triad can be observed in any modality, it is more commonly seen on CT (77.8%), as reported by Lassandro et al. vs. 14.8% and 11.1% on abdominal film and ultrasound, respectively, concluding that X-ray and ultrasound are also suitable modalities for screening [13]. At the same time, CT is better for an in-depth view and is considered the gold standard [9,13]. Selectively, magnetic resonance cholangiopancreatography can be used in inconclusive CT images [9].

Managing gallstone ileus centers around relieving the obstruction by retrieving the gallstone. Fluid and electrolyte imbalances should also be addressed, especially in patients with comorbidities like our hypertensive elderly patient who presented with azotemia, highlighting the disease's progression [2,5]. Obstruction causes vomiting, which subsequently leads to electrolyte imbalance, dehydration, and eventually azotemia [5].

Not all gallstone ileus cases require surgical intervention; most gallstones smaller than 2 to 2.5 cm may pass spontaneously [1]. In our patient, the gallstone was 3.5 cm. Surgical management options for gallstone ileus include (1) simple enterolithotomy; (2) a one-stage procedure involving enterolithotomy, cholecystectomy, and fistula closure; and (3) a two-stage procedure where enterolithotomy is performed initially, followed by cholecystectomy later [5]. We approached our patient with the last-mentioned option; however, the patient refused the cholecystectomy, as the symptoms had entirely resolved. Although there is controversy regarding the timing of cholecystectomy, the authors agree on a one-stage procedure for highly selected cases due to its higher association with morbidity. Due to our patient's presentation, we opted to reserve cholecystectomy for a more optimal status [6]. In a review, Inukai suggests the surgical method to be based on an impaction site, two stages of surgery at the small intestine level while preserving a one-stage surgery for impaction on other sides while considering the patient’s general status and presentation [2].

MRCP was performed during a follow-up 12 months after the surgery, which showed the closure of the fistula track between the first part of the duodenum and the gallbladder.

## Conclusions

Gallstone ileus is a challenging condition that demands quick identification and decisive intervention. In this case, the choice between a one-stage and a two-stage surgery wasn't just about following protocol; it was about weighing the immediate need to clear the obstruction against the risks of a more invasive procedure. Opting for a two-stage approach allowed us to handle the immediate problem and keep our options open for later. It's a reminder that in complex cases like these, sometimes, the best strategy is to take it step by step, adjusting the plan as the situation evolves.

## References

[1] Clavien PA, Richon J, Burgan S, Rohner A (1990). Gallstone ileus. Br J Surg.

[2] Inukai K (2019). Gallstone ileus: a review. BMJ Open Gastroenterol.

[3] Martin F (1912). Intestinal obstruction due to gall-stones. Ann Surg.

[4] Bouveret L (1896). Stenose du pylore, adherent a la vesicule calculeuse [Article in Corsican]. Rev Med.

[5] Nuño-Guzmán CM, Marín-Contreras ME, Figueroa-Sánchez M, Corona JL (2016). Gallstone ileus, clinical presentation, diagnostic and treatment approach. World J Gastrointest Surg.

[6] Cooperman AM, Dickson ER, ReMine WH (1968). Changing concepts in the surgical treatment of gallstone ileus. A review of 15 cases with emphasis on diagnosis and treatment. Ann Surg.

[7] Beltran MA, Csendes A (2005). Mirizzi syndrome and gallstone ileus: an unusual presentation of gallstone disease. J Gastrointest Surg.

[8] Halabi WJ, Kang CY, Ketana N (2014). Surgery for gallstone ileus. A nationwide comparison of trends and outcomes. Ann Surg.

[9] Chang L, Chang M, Chang HM, Chang AI, Chang F (2018). Clinical and radiological diagnosis of gallstone ileus: a mini review. Emerg Radiol.

[10] Alibegovic E, Kurtcehajic A, Hujdurovic A, Mujagic S, Alibegovic J, Kurtcehajic D (2018). Bouveret syndrome or gallstone ileus. Am J Med.

[11] Zaliekas J, Munson JL (2008). Complications of gallstones: the Mirizzi syndrome, gallstone ileus, gallstone pancreatitis, complications of "lost" gallstones. Surg Clin North Am.

[12] Qian W, Soares J, Jayewardene ID, Peck N (2024). Bouveret syndrome preceding classical gallstone ileus: a rare presentation of a cholecystoduodenal fistula. J Surg Case Rep.

[13] Lassandro F, Gagliardi N, Scuderi M, Pinto A, Gatta G, Mazzeo R (2004). Gallstone ileus analysis of radiological findings in 27 patients. Eur J Radiol.

